# PREDICTORS OF ATTRITION IN THE MULTI-ETHNIC 1FLORIDA ADRC CLINICAL CORE

**DOI:** 10.1093/geroni/igz038.1733

**Published:** 2019-11-08

**Authors:** Shanna L Burke, Warren Barker, Monica Rosselli, Miriam Rodriguez, Carolina Robayo, Cesar L Chirinos, Raquel Behar, Maria T Greig-Custo

**Affiliations:** 1 Florida International University, Miami, Florida, United States; 2 Mount Sinai Medical Center, Miami Beach, Florida, United States; 3 Florida Atlantic University, Department of Psychology, Florida Atlantic University, Department of Psychology, Florida, United States; 4 Albizu University, Miami, Florida, United States; 5 Wien Center for Alzheimer’s Disease and Memory Disorders, Miami Beach, Florida, United States

## Abstract

Understanding predictors of attrition can position researchers to increase retention efforts and focus on preventing attrition. Attrition, or dropout of participants during a study prior to completion, can threaten the internal and external validity of a study’s findings. Data from the 1FloridaADRC Clinical Core was analyzed, and included 271 participants within a two-year follow-up window, of which 216 (79.7%) were retained. T-tests and chi-square analyses were used to determine if a number of demographic, clinical, acculturation, and neuroimaging predictors were associated with attrition. The participant cohort included: 85% with cognitive impairment; 60% Hispanic; 42% over the age of 75; and 62% female. Predictors of greater attrition included: age over 75 years (p< .003); cognitive diagnosis of MCI or dementia (p< .01); and lower scores on the Mini-Mental Status Exam (p<.04), the Hopkins Verbal Learning Test (HVLT) immediate (p< .02), and delayed (p<.002) Higher total score on the Neuropsychiatric Inventory Questionnaire (p<.06), endorsement of night time behaviors (p<.05) and greater hippocampal atrophy (p<.02) were also predictive of attrition. Hispanic ethnicity was not a predictor of attrition, as retention was 81% for Hispanics versus 79% for non-Hispanics. However, among Hispanic participants, English acculturation measured by the Bidimensional Acculturation Scale for Hispanics was lower for those who dropped out (t=2.8; p=.006).

